# Swarm formation morphing for congestion-aware collision avoidance

**DOI:** 10.1016/j.heliyon.2021.e07840

**Published:** 2021-08-18

**Authors:** Jawad N. Yasin, Mohammad-Hashem Haghbayan, Muhammad Mehboob Yasin, Juha Plosila

**Affiliations:** a Autonomous Systems Laboratory, Department of Future Technologies, University of Turku, Vesilinnantie 5, 20500 Turku, Finland; b Department of Computer Networks, College of Computer Sciences & Information Technology, King Faisal University, Hofuf, Saudi Arabia

**Keywords:** Multi-agent systems, Autonomous swarm, Congestion awareness, Swarm intelligence, Collision avoidance, Formation maintenance

## Abstract

The focus of this work is to present a novel methodology for optimal distribution of a swarm formation on either side of an obstacle, when evading the obstacle, to avoid overpopulation on the sides to reduce the agents' waiting delays, resulting in a reduced overall mission time and lower energy consumption. To handle this, the problem is divided into two main parts: 1) the disturbance phase: how to morph the formation optimally to avoid the obstacle in the least possible time in the situation at hand, and 2) the convergence phase: how to optimally resume the intended formation shape once the threat of potential collision has been eliminated. For the first problem, we develop a methodology which tests different formation morphing combinations and finds the optimal one, by utilizing trajectory, velocity, and coordinate information, to bypass the obstacle. For the second problem, we utilize thin-plate splines (TPS) inspired temperature function minimization method to bring the agents back from the distorted formation into the desired formation in an optimal manner, after collision avoidance has been successfully performed. Experimental results show that, in the considered test scenario, the proposed approach results in substantial energy savings as compared with the traditional methods.

## 1 Introduction

The study of the behavior of a system comprising a large number of autonomous agents that interact amongst themselves as well as the environment is generally classified as swarm robotics [1], [2]. The compliance of comparatively simple control routines serves as the underlying foundation of swarm robotics, i.e., a multi-agent system. The agents, individually or collaboratively, obey the routines while utilizing their respective on-board perception sensors and communicating with other neighboring agents [3]. Study of swarms of UAVs (drones) has seen a rising interest from the research community due to their integration in diverse application fields, such as transportation [4], atmospheric research [5], surveillance [6], entertainment [7], and mapping in GPS-denied environments [8], due to their ability to work in a collaborative and cooperative manner [9]. Navigation of a swarm of agents introduces several research challenges. Among these, the two most significant ones are formation maintenance and collision avoidance [10]. Collision avoidance systems are responsible for guiding an autonomous agent in order to safely and reliably avoid potential collisions with other agents in the swarm as well as with other objects in the environment [11]. For agents to be fully autonomous, not having to bank on a central server increases the robustness of the system, and therefore the ability to locally sense and avoid objects in the environment becomes of greater importance. This local collision avoidance ability becomes even more vital in a multi-agent system where multiple agents are collaborating together to achieve a desired task, for instance navigation to the desired destination [12]. Moreover, formation maintenance algorithms are responsible for guiding the agents to maintain a certain/desired shape, i.e., location of every agent is defined with respect to other agents in the swarm [13]. Formation control approaches can be divided into the following three categories, i.e., leader-follower based approach [10], [14], behavior-based approach [15], [16], and virtual structure based approach [17], [18]. Among the aforementioned methodologies, the leader-follower based approach is more common pertaining to its ease of analysis, robustness, scalability, and implementation [13], [19].

Reducing energy consumption to increase mission life is another important research area in swarm robotics, focusing on a diverse set of topics, such as efficient decision making [20], minimization of traveling distance [21], energy efficient communication for swarm robot coordination [22], decreasing the usage of ranging sensors [23], and autonomous recharging [24]. In this paper, we present a novel approach to avoid congestion that may occur due to the overpopulation in either of the available gaps between the obstacles, resulting in delays and consequently higher energy consumption of the agents as well as the swarm as a whole. This is handled in two phases: the first is the estimation phase for finding the optimal distribution of the agents into sub-groups for formation morphing while bypassing the obstacles, and the second is bringing the agents back into the intended formation shape, i.e., a reformation step. In the estimation phase, as soon as the obstacles are detected, the leader utilizes the coordinate, velocity, and trajectory information of the agents in the swarm to estimate and find the optimal distribution of the agents through the available gaps between the obstacles. Once the obstacle avoidance is successfully achieved, the agents are brought back into the intended formation shape by utilizing a temperature minimization function, that is inspired by the thin-plate splines technique.

The rest of the paper is structured as follows. Related work is covered in Section 2. Preliminaries and the problem formulation are described in Section 3. Section 4 covers the proposed approach in detail. Experimental setup is given in Section 5. Simulation results and the related discussion are covered in Section 6. Finally, Section 7 provides the concluding remarks.

## 2 Related work

In relation to the existing literature, the proposed approach is most related to the energy-efficient path planning or collision avoidance and time criticality. Some path planning methods rely on a prior map to pre-process the data for building navigational paths. For instance, the vector field approach [25] and Probabilistic Roadmap (PRM) [26] essentially build the path by searching the connectivity graph generated by randomly sampling the map. In the aforementioned methods, part of the processing is performed offline to facilitate the online processing. However, these methods are still computationally expensive, due to the fact that online processing is still required to go through the graph to find the path [27].

On the other hand, sampling-based methods, like Batch Informed Trees (BIT) [28] or Rapidly exploring Random Tree (RRT) based methods [29], generate paths by taking random samples from the graph and connecting them. Although such methods have shown potential for generating paths faster due to their ability to manipulate large scale maps, they are still computationally heavy especially in complex environments. Therefore, the ability to find a path within given time constraints cannot be guaranteed.

Moreover, Multilevel Sub-graph Patrolling (MSP) algorithms [30], [31] divide the map into a number of zones and allocate different zones to the individual robots. These approaches rely on prior information of the environment and computations are performed based on that global information by a central computing unit. Therefore, any change in the environment may affect the harmony of the system's optimization as the predefined trajectories may not be optimal for such changes [32].

The work presented in [33], [34] are the closest related to our proposed approach. However, the congestion aware solution that is beneficial for the swarm as a whole is still not addressed. In [33], the authors propose a methodology for controlling the velocities of the robots in case of jams due to over-crowding in a queue. They propose a robot behavior regulation rule and integrate it with adaptive cruise control of the vehicle/robot to adjust the speed in case of high density of robots in a zone to avoid jamming. In [34], the Spread-Out Localization-Space Trails (SO-LOST) algorithm is proposed. The approach is focused on reducing the interference between the agents by using the SO-LOST algorithm for generating spatially-separated trails for each robot, one from the home location to the destination and the other from the destination to the home location. The authors in [35] present a methodology to prevent congestion among robots, while performing different tasks, based on a probabilistic reservation model. The model sequentially approximates for each robot the time to reach the destination in a predefined priority based order. The schedules are implemented once all the robots have executed their respective plans in a sequential manner. However, the presented work does not take into account the overall efficient solution for the swarm as a whole.

## 3 Problem formulation

Our previous works have focused on: (1) energy-efficient formation morphing by systematic integration of formation control and collision avoidance for formation-collision co-awareness and the use of non-rigid mapping by utilizing a thin-plate splines (TPS) based algorithm to minimize deformation in the swarm [10]; (2) reducing the energy consumption owing to sensor(s) usage in the swarm by introducing the concepts of translational coordinates based navigation and adaptive consciousness in the agents [23], [36]; and (3) dynamic formation reshaping for collision avoidance while passing through the available gaps between the obstacles without slowing down [37].

**Figure 1:**
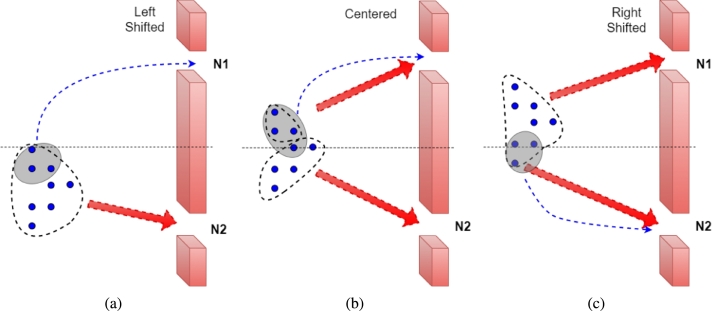
Illustration of encountered obstacles (a) extending more towards the left w.r.t. the swarm, (b) centered with the swarm, i.e., left or right edges of the obstacle lie at same distance, (c) extending more towards the right w.r.t. the swarm.

**Figure 2:**
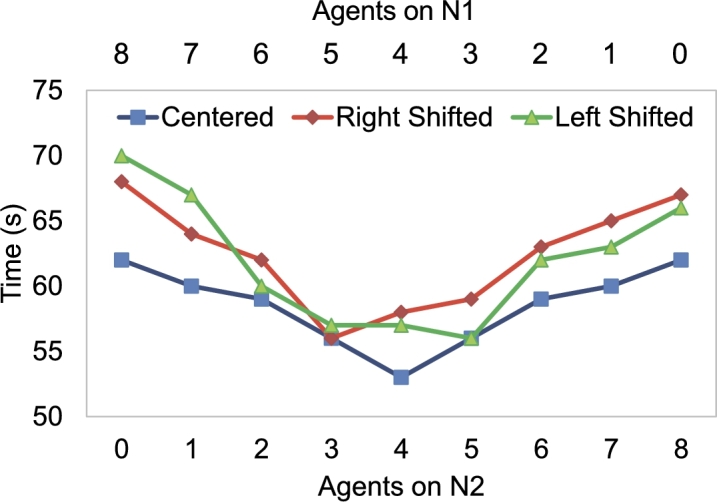
Obstacle's location w.r.t. the swarm: Centered = Obstacle's center is in the same as the swarm's center, Left Shifted = Obstacle is shifted towards left side from the center of the swarm, Right Shifted = Obstacle is shifted towards right side from the center of the swarm.

Current approaches of minimizing the traveling distance while performing collision avoidance maneuvers may adversely impact the energy efficiency of the swarm owing to congestion on narrow pathways. This serves as a key motivation for the approach proposed in this paper. Fig. 1 illustrates the scenario where individual drones choose the shortest path while avoiding an obstacle. If the encountered obstacle is not aligned with respect to the swarm's center, as illustrated in Fig. 1(a) and 1(c), more agents will prefer one path (say N1) over the other (say N2) causing congestion on that path. In the said figure, the group of agents that will navigate towards N1 or N2 are grouped by encircling them in a dotted circle for illustration purposes. The resulting congestion or over-population causes an increase in overall time for the swarm to reach the destination and an associated increase in the energy consumption of the agents. The congestion-aware distribution methodology proposed in this paper works by forcing some of the agents (encircled by a gray circle) to opt for the longer distance in order to find the optimal solution for the swarm. As shown in Fig. 2 and elaborated in Section 5, choosing collision avoidance maneuvers that focus on time minimization of the swarm as a whole is a more effective method.

**Figure 3:**
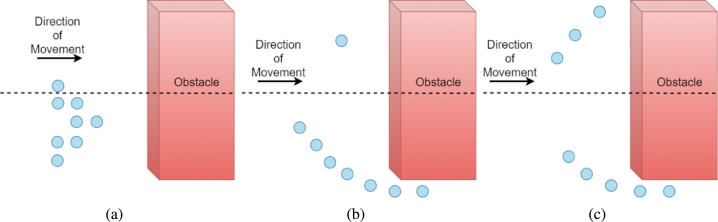
Swarm encountering obstacle (a) the initial configuration, (b) shortest path swarm distribution, (c) swarm distribution utilizing the proposed approach.

When a swarm of autonomous drones encounters an obstacle(s), the agents take local decisions to perform collision avoidance maneuvers. Fig. 3 shows an example scenario of a swarm with eight agents avoiding an obstacle using the two different approaches. The initial configuration is illustrated by agents in “blue” (Fig. 3(a)). The cases illustrated are as follows: 1) swarm in distribution while performing collision avoidance using shortest path approach (Fig. 3(b)), 2) the distribution of the swarm agents with the proposed approach (Fig. 3(c)). The apparent answer to the collision avoidance problem is for each drone to select the nearest end of the obstacle and go round the corner as the optimum route, namely: the shortest path approach [38]. As exemplified in the aforementioned figure, the optimal formation disturbance for the swarm may not follow the shortest path rule, for example in Fig. 3(b) if each agent moves towards the edge of the obstacle with respect to its own coordinates to follow the shortest path, it will take more time for the swarm to bypass the obstacle since the agents will have to slow down to avoid congestion from neighboring agents. On the other hand, if the agents follow the proposed optimal morphing configuration, illustrated in 3(c), the swarm distribution is done in a manner to minimize the overall time penalty. In order to avoid the congestion and resultant delays, some of the agents are directed to choose longer routes in order to minimize the overall time taken by the swarm to pass the obstacle.

The main motivation behind the proposed approach comes from the hypothesis that the selection of avoidance route may apparently be the shortest path whereas it may not be optimal when considering a time-aware objective function. Therefore, the problem is how to avoid the obstacle in an efficient way, i.e., to minimize the overall time required by the swarm to perform an avoidance maneuver, without increasing the velocity significantly, i.e., aggressive acceleration. For example, for an individual agent selecting the nearest edge to bypass the obstacle may be optimum, but for the swarm as a whole, it may not be. This is due to the fact that delays occur when the swarm has to deviate from its original trajectory to either avoid an obstacle or go through the available gap between the obstacles and the agents have to slow down, wait, or allow for other neighboring agents to go ahead or merge in the queue as shown in Fig. 3(b). Now it is important to note here that if an obstacle, assuming the obstacle is in detection range and both corners are visible, clearly extends towards one side of the swarm does not mean that going for the shortest path will provide optimal results, i.e., the minimum time for the last agent to pass through. Here we are calculating the time from when the obstacle is detected till the last agent passes the center of the obstacle, which is our cost function.

To support this claim, we investigate three different scenarios where a swarm faces an obstacle in its way: the first scenario is when the obstacle is in line with the center of the swarm, the second is when the obstacle is to the left of the swarm, and the third is when the obstacle is to the right of the swarm. Fig. 2 shows the timing result of these three scenarios. Here, the agents are divided into two groups, namely: Group N1 deviates towards the left corner and Group N2 deviates to the right corner as shown in Fig. 5. In this case, the swarm is composed of 8 agents, in a nested V-shaped formation as shown and the results are reported for: 1) *Centered*, when the obstacle's center is inline with the center of the swarm, the optimal result obtained is when *N1* and *N2* both have 4 agents, it took the swarm 53 sec to pass the obstacle (as shown in Fig. 2), 2) *Left Shifted*, when the obstacle's center is shifted to left side w.r.t. the swarm's center, the optimal result was acquired with 3 agents in *N1* and 5 agents in *N2*, and 3) *Right Shifted*, when the obstacle's center is shifted to right, the optimal timing for bypassing the obstacle is obtained, i.e., $t_{min}=56$ s with 5 agents in *N1* and 3 agents in *N2*.

It is important to note here that even though it might be possible to reduce the delays by accelerating aggressively to minimize the time delay, however, it has an adverse effect on the power consumption of the agent as the minimum power requirement changes [39]. Therefore, in the performed simulations, the agents maintain a nominal velocity of $v_{i}$ whenever possible and are allowed to accelerate/decelerate only gradually.

## 4 Proposed approach

**Figure 4:**
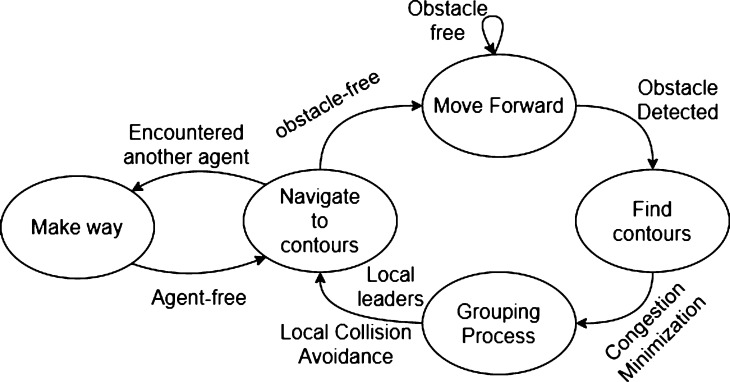
Simplified finite state machine of the congestion aware collision avoidance.

**Figure 5:**
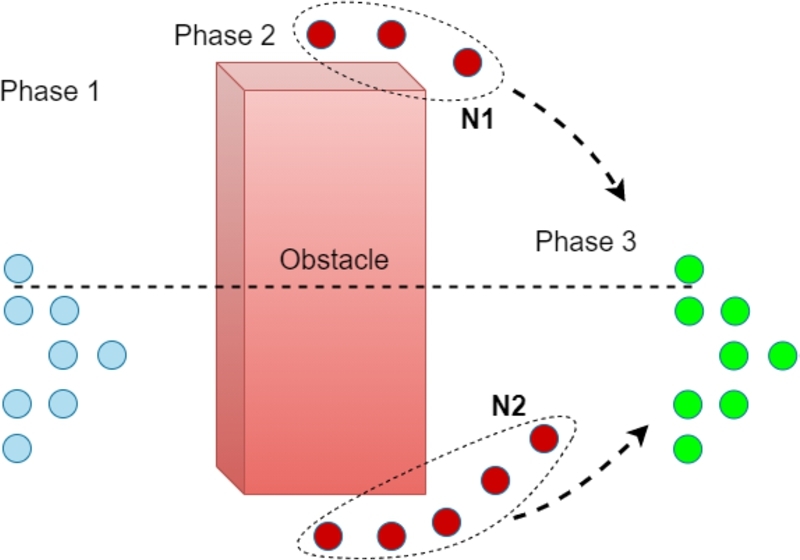
Illustration of 3 phases: Phase 1) the initial phase, Phase 2) system at highest disturbance point, Phase 3) the convergence phase.

In this section, we describe the proposed Swarm Formation Morphing for Congestion Aware Collision Avoidance algorithm for a swarm of autonomous agents; a simplified system flow diagram is given in Fig. 4. The overall strategy is to combine the optimal formation morphing, in the presence of obstacles, to avoid overpopulation and the reformation mechanism that facilitates efficient navigation of the swarm, see Fig. 5. In the optimal formation morphing for avoidance maneuver, based on the number of obstacles, the population factors are evaluated for the agents of the swarm. Then using the population factor and the time these factors require for avoidance completion, agents are divided into different sets of groups. These set of groups of agents nominate their own respective local leaders of the groups. Once the obstacle avoidance is successfully completed, the swarm formation is in a highly disturbed state. Next, in the convergence phase, thin-plate splines based reformation methodology is used for bringing the agents back into the intended formation. Here, based on the position vectors, agents are mapped onto the desired formation positions in an optimal manner.

Now, the detailed working of the proposed approach is presented. It is assumed that, initially the swarm in flying in the desired formation shape and the communication amongst the agents is already established. Algorithm 1 highlights the overall routine of the proposed approach. This top-level algorithm is executed by each agent locally, by utilizing its onboard processing unit. Algorithm 1 starts by checking and setting up the leader for the swarm and then proceeds to connect the follower agents with their respective leaders if it has not been established yet (Line 2). Then based on the current state, i.e., position, of each agent in the swarm, the Target_Shape of the swarm is decided (Line 3) and target coordinates for each agent are calculated. Here Target_Shape represents the intermediate shape of the formation to be attained at the end of each iteration on the way to reach the desired final formation.

**Algorithm 1:**
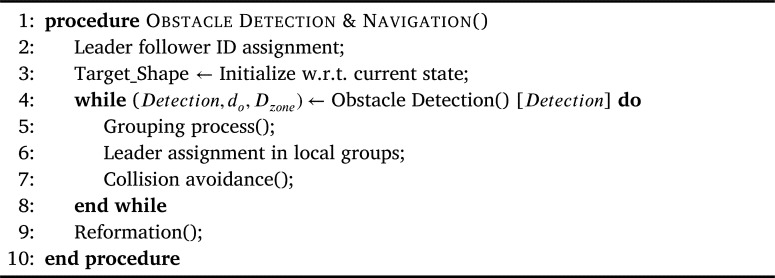
Global Routine.

After initialization, the main loop begins (Lines 4-8), where *Obstacle Detection* is the first procedure that is executed (Line 4). In case, an obstacle is in the detection range, a certain set of rules are executed (Lines 5-7). First the procedure *Grouping Process()* is called. This procedure is responsible for determining the optimal formation morphing configuration. By utilizing the information provided by *Obstacle Detection()*, i.e., the number of obstacles, this procedure calculates the population factors and the respective times they require to go past the obstacle, then the optimal solution is chosen. Next, based on the aforementioned calculations and leader determination from the *Grouping Process*, leader assignment is done (line 6). Then the procedure *Collision Avoidance* is called to guide the agents reliably and safely away from the potential collisions (Line 7). Finally, the reformation procedure based on thin-plate spline is called to bring the agents back into the desired formation (Line 9). Its effect is only significant in case the formation has been distorted because of collision avoidance. The utilization of point set registration in the reformation process is of particular significance as it is vital to do the mapping between the current and the expected shapes optimally and swiftly.

### 4.1 Obstacle detection

**Figure 6:**
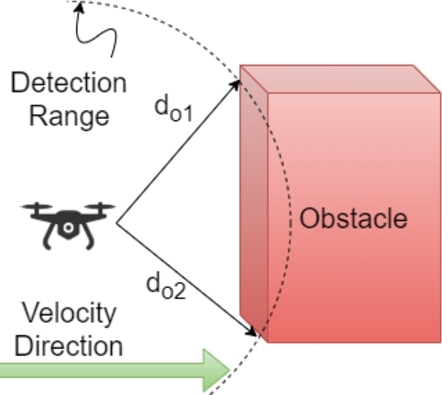
Obstacle Detection.

The pseudo-code for this procedure is specified in Algorithm 2, in which the agent scans for the presence of any objects continuously and the moment an object is detected by the onboard sensor system, the Detection flag is set to *True* (Lines 2-3). Then based on the sensor's feedback, the calculation of the detected object's parameters is done, i.e., the distance at which the object is detected and the angle to it (Line 4), as shown in Fig. 6.

**Algorithm 2:**
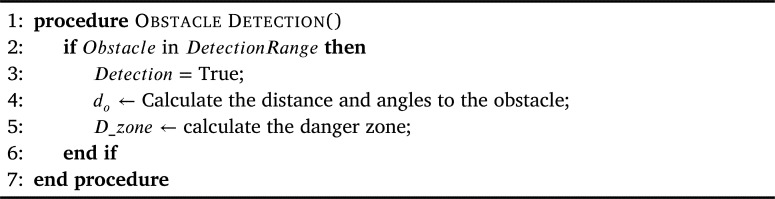
Obstacle Detection.

Considering the velocity at which the agent itself is traveling and the distance to the detected obstacle, the danger zone is defined, beyond which the collision is imminent and can not be avoided (Line 5). The danger zone is defined to adjust the velocities of the agents appropriately based on the stopping distance of the agent, as elaborated in the equations below. We know the distance to the obstacle and the velocity of the agent, then the time to potential impact $t_{imp}$ is calculated by:

(1) $$t_{imp}=d_{oi}/v$$

where $d_{oi}$ is the distance to the object(s) and *v* is the agent's velocity. The distance that an agent will travel after detecting the obstacle and before it comes to a complete halt, called the stopping distance, is composed of two parts. Firstly, the distance it continues to travel while obstacle detection and avoidance algorithm is executing; this duration is called the reaction time and the distance so traveled is called the reaction distance. Secondly, the distance it covers while decelerating to a complete stop, referred to as the braking distance. Therefore, the stopping distance is computed as follows:

(2) $$d_{s}=d_{r}+d_{b}$$

Where $d_{s}$, $d_{r}$, and $d_{b}$ are the stopping distance, reaction distance, and braking distance respectively. Braking distance ($d_{b}$) and reaction distance ($d_{r}$) are calculated as follows:

(3) $$d_{b}=v^{2}/2gc_{d}$$

(4) $$d_{r}=vt_{c}$$

Where *g* is the gravitational constant, $c_{d}$ is the air drag coefficient, and $t_{c}$ is the time it takes to compute or react.

### 4.2 Grouping process

In this process, specified in Algorithm 3, the leader approximates by simulating the time to avoid the obstacle(s) for all the combinations of the agents by utilizing their respective velocities and coordinates. The number of obstacles (*{obsSet}*), in the vicinity, is used to calculate the population factor set (*{popfacSet}*), Line 2 in Algorithm 3. For instance, if there is one obstacle, then the population factor is two, since there are two possible paths for the agents to navigate through. The available number of population factors can be defined by the following relation:

(5) $$pf_{i}=obs_{i}+1$$

where $pf_{i}$ is the number of population factor and $obs_{i}$ is the number of obstacles. Then based on the population factor set and the penalty of time, i.e., the group configuration that requires the minimum amount of time to pass the obstacle, the agents are divided into different groups (*{groupSet}*) is calculated (Line 3), as illustrated in Fig. 3. The agents' distribution into the *{groupSet}* is done based on the following metric:

(6) $$\tau =\frac{\Sigma D_{i}}{v_{i\in J}}$$

*τ* is the overall time, Σ*D* is possible paths based summation of the distance from agent's current location to estimated avoidance location, $v_{i}$ is the velocity of the agents, *J* is the number of agents. Where $D_{i}$ is

(7) $$D_{i}=f_{Gi}({\bar{d}}_{g\in G_{i}})$$

**Algorithm 3:**
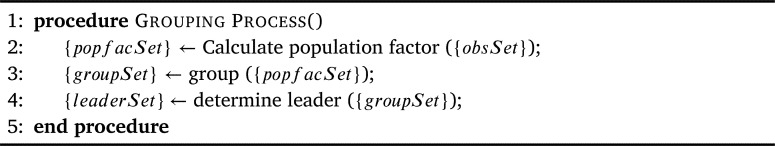
Grouping Process.

$f_{Gi}$ is the geometric median of the group $G_{i}$, $\bar{d}$ is the average distance of the agents from each other, $G_{i}$ is the *groupSet*. The relation between the $\bar{d}$ to population factor, i.e., $pf_{i}$ is

(8) $$\bar{d}\propto 1/\bar{pf_{i}}$$

and the relation between $pf_{i}$ and safe_distance, i.e., the defined safe distance to be maintained amongst the agents and the velocities is given as

(9) $$pf_{i}\propto 1/\text{safe}\_\text{distance}$$

(10) $$v_{i}\propto 1/pf_{i}$$

Afterwards, *{leaderSet}* gets the leaders determined from the respective calculated groups, i.e., *{groupSet}* (Line 4). In each group set, electing group leader within that group provides the same functionality as that of the swarm leader, as it may be required to divide the group into further sub-groups to avoid congestion, in case the group encounters further obstacles.

### 4.3 Collision avoidance

The pseudo-code, in Algorithm 4, describes the collision avoidance procedure. This procedure is executed when the obstacle is detected and the calculated distance and angles suggest that continuing the trajectory will lead to a collision. It starts by checking if there were multiple obstacles detected (Line 3). In case, the detected obstacles are more than one, the available gap between the obstacles is then calculated (Line 4). If the gap is greater than the defined minimum safe distance (minimum allowed distance on either side of the agent plus agent's dimensions), the agent is aligned to navigate through the obstacles (Lines 5-6). Otherwise, the obstacles are enveloped as one obstacle, and path planning is done accordingly to bypass a single obstacle. In case only one obstacle was detected initially, path planning is performed, for a single obstacle, to bypass the obstacle (8-10). For aligning the agent to navigate through the gap between the obstacles and path planning, we utilized and implemented the technique presented in [40]. If the distance to the obstacle is no longer in the detection range, the control is returned to the overall routine by resetting the *Detection* flag to *False*.

**Algorithm 4:**
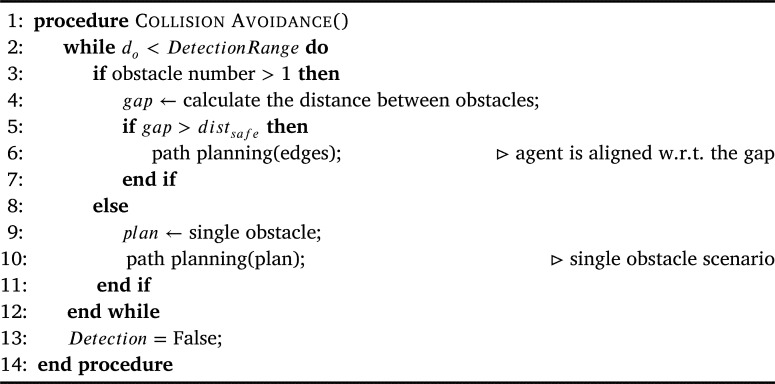
Collision Avoidance.

### 4.4 Reformation

We take inspiration from the technique presented in [10] and base the reformation function by utilizing point set registration [41], [42] that is based on a well known technique used to data interpolation and smoothing issues, i.e., thin-plate splines (TPS) [43]. The amount by which the formation is distorted is assessed by the energy function as shown in Eq. (6).

(11) $$E_{TPS}(f)=\sum_{i=1}^{n}||x_{i}-f(v_{i})|{|}^{2}+\lambda \iint [{\left(\frac{\partial^{2}f}{\partial x^{2}}\right)}^{2}+2{\left(\frac{\partial^{2}f}{\partial x\partial y}\right)}^{2}+(\frac{\partial^{2}f}{\partial y^{2}})]dxdy$$

Where $E_{TPS}$ is the energy function and *λ* is the scaling factor. Mapping a set of points to the corresponding point sets while keeping the intended *formation* under consideration, is handled by the integral part of the equation. Since, our intention is to only map one set of points over the other and without considering the distorted shape of the swarm, therefore in order to map the closest points we set the scaling factor (*λ*) to zero. This reduces the Eq. (11) as follows:

(12) $$E_{TPS}(f)=\sum_{i=1}^{n}||x_{i}-f(v_{i})|{|}^{2}$$

It is important to note here that the TPS algorithm is not generating the control velocities, but generating the next/future coordinates for the agents to navigate to. Then based on those, the agents draw their respective tangents and adjust their respective velocities.

The overview of the TPS-based reformation function is provided in Algorithm 5. This procedure starts by computing the next, i.e., the future, location of each agent based on the present coordinates of the agents (Line 3). Then agents are assigned new coordinates based on the determined new location (Line 4). Then, to perform reformation as optimally as possible, these determined values are passed to the temperature minimization function based on TPS, for bringing the agents to their respective updated locations as optimally as possible (Line 5). As soon as every agent has reached its respective new location or coordinates, the control is returned to the overall routine.

**Algorithm 5:**
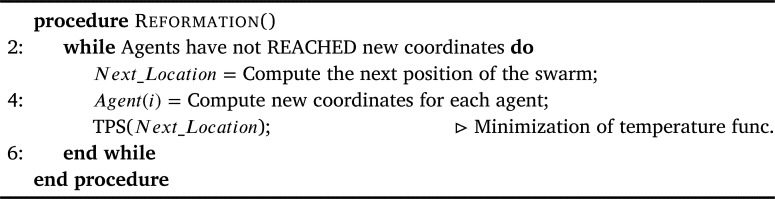
Reformation.

The main issue to discuss about the complexity is the convergence of the algorithms. As it can be noticed in Algorithm 1, the algorithm repeats the evaluation and action process until an obstacle is in the range of the swarm agents. Most parts of other algorithms are simple conditional statements in the form of *if-then-else* with complexity O(1) and the nodes are performing in parallel the computations individually. Moreover, some parts of the algorithm can be executed pro-actively, such as the pre-evaluation part in the collision avoidance algorithm. The complexity for the grouping process is O(n), where *n* is the number of nodes in the swarm. Here, the leader has to do estimation analysis to find the optimal distribution solution for all nodes. The complexity of the proposed approach relies on the presented algorithms as follows: Algorithm 1, Algorithm 2, Algorithm 4, and Algorithm 5 have the complexity of O(1). Whereas, Algorithm 3, i.e., grouping process, has the complexity of O(n), where *n* is the number of nodes in the swarm. It is worth mentioning that since the algorithm will be triggered only when the swarm is facing an obstacle and when the swarm is progressing, the velocities of the nodes toward the mission path never reaches zero. It effectively means that the algorithm never enters an endless loop and will end when the swarm passes the obstacle, even when the algorithm is not computed entirely. In such cases, i.e., when the algorithm does not have enough time to optimize in the base applicable way, the swarm will avoid and pass the obstacles with some penalty of power, i.e., some expected power saving by the algorithm will be lost.

## 5 Experimental setup

For simulating agents in the swarm, point mass particle model and the point mass particle's equations of motion are utilized. SwarmLab, a MATLAB Drone Swarm Simulator [44], is used for evaluating the proposed idea. The communication module for each drone, to evaluate the power, is Legacy Digi XBee-Pro S1 802.15.4, owing to the possibility of connecting large number of devices and its longer transmission range, i.e., 100 m urban/indoor environment, and data rate of 250 kbps. The power consumption in Transmitting Mode and Receiving Mode is 710 mW and 182 mW respectively [45]. The consumption due to the use of ranging sensor, for sense and avoid function, is evaluated based on the consumption of Velodyne Puck LITE, i.e., 8 Watts typically [46].

Assumptions and initial conditions considered in this work are defined as follows:

1. all drones are at the same altitude
2. agents accelerate or decelerate linearly
3. the position vectors of the agents are obtained by utilizing on-board localization techniques
4. the communication channel is ideal, i.e., without information loss and delays
5. the computational or reaction time of the agent is considered to be negligible, and the reaction distance ($d_{r}$) is zero.

## 6 Simulation & results

**Figure 7:**
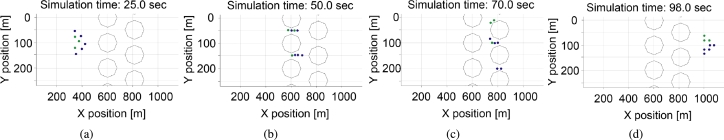
Simulation snapshots. (a) time = 25 s of the simulation. (b) time = 50 s of the simulation, swarm navigating through the obstacles. (c) time = 70 s, groups divided into further subgroups upon encountering another set of obstacles (d) towards end of simulation.

**Figure 8:**
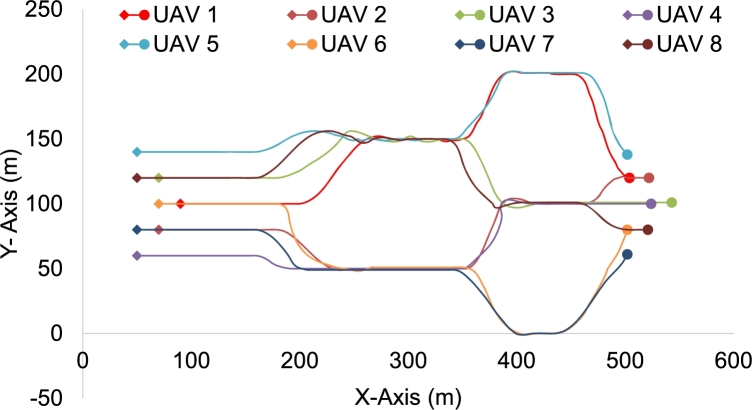
Trace of Overall Movement of the Agents. Here starting locations of all the agents are represented by the diamond shape (⋄) and the final locations of the agents are represented by a dot shape (•).

The mission starts with agents already in a defined nested V-shaped formation moving towards the destination in an open environment. Fig. 7(a) shows the disturbed formation at *Simulation time* = 25 s when the obstacle has been detected and the agents have started deviating to avoid the potential collision, i.e., the first layer. In Fig. 7(b), the formation disturbance is at maximum, the agents, grouped to avoid congestion on either side (*N1*, *N2*)^1^ while keeping the minimum safe distance from the obstacle and from each other, are bypassing the obstacle. As soon as the agents pass the obstacle, the second set of obstacles is detected by both groups locally. As shown in Fig. 7(c), the agents divide themselves locally into further sub-groups (we call *N11*, *N12* and *N21*, *N22* for subgroups from *N1* and *N2* respectively) to avoid congesting either side. The agents in *N12* and agents in *N21* are navigating towards the same route to bypass the obstacles. In this case, the agent farthest ahead takes precedence and in a similar manner, they merge to form a queue formation to navigate through space between the obstacles. Fig. 7(d), shows the swarm's reformation once there is no obstacle in the detection range of the agents. The trace for the overall movement of the agents throughout the mission is shown in Fig. 8, where morphing of the formation is visible, through a forest-like environment. In the figure, the starting points/positions of the agents are denoted by a “diamond” shape and the final positions are denoted by a solid circle.

**Figure 9:**
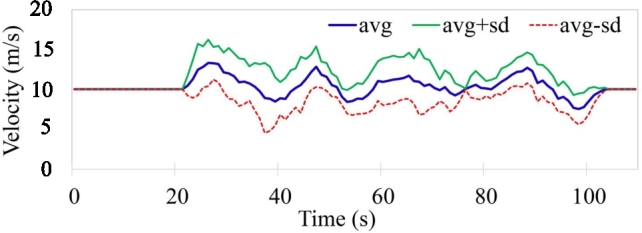
Report of the average Velocity, average ± standard deviation of the swarm.

**Figure 10:**
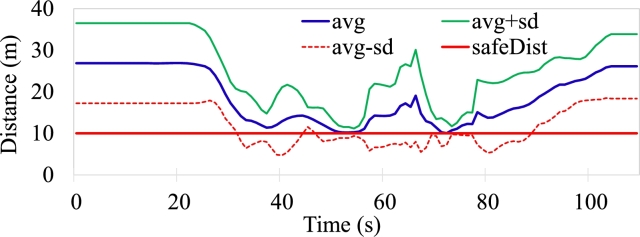
Average distance maintained by the agents from mission start to obstacle avoidance to mission end, average ± standard deviation of the swarm.

Fig. 9 shows the average velocity of the swarm as a whole, Fig. 10 shows the average distance maintained by the agents in the swarm; the standard deviation of the velocity and distance is also plotted for reference. The non-aggressive variance in the average velocity is due to the fact that agents have to slow down to provide enough space for another agent, deviate to bypass the obstacle, slow down if the agent in front is slowing down (Fig. 7(b)), and maintain tight queue formation while going through obstacles.

In Fig. 10, the solid red line shows the defined minimum safe distance in the performed experiment, which is the minimum distance agents maintain with each other in the disturbance phase, i.e., while performing collision avoidance. The peak at around *t* = 43 s, is due to the change in immediate respective leaders due to the *grouping process*, Fig. 7(b). The second sudden change in the average distance maintained, at *t* = 66 s, is due to the encounter with the second set of obstacles and the previous groups getting divided into further groups and the resultant change in immediate leaders through *grouping process*. From around *t* = 80 s onwards, the agents are coming back into the desired formation shape and maintaining the formation defined inter-agent distance. There is a negligible error of 0.8 m in the average distance maintained between the agents when reformation is completed.

To evaluate the effectiveness of the proposed technique, we implemented the well-known techniques of shortest path based formation morphing and collision avoidance (EFMCA) and leader-follower formation (IOAA) presented in [10] and [14] and set the results side by side with the obtained results from the proposed technique in the similar experimental setup. Here we evaluate the performance in two different setups: normal and complex. The normal complexity scenario is illustrated in Fig. 1, where the swarm encounters one layer of obstacles, i.e., three obstacles parallel to each other, and the high complexity scenario is shown in Fig. 7, where the swarm encounters nested obstacles and navigates through them.

**Figure 11:**
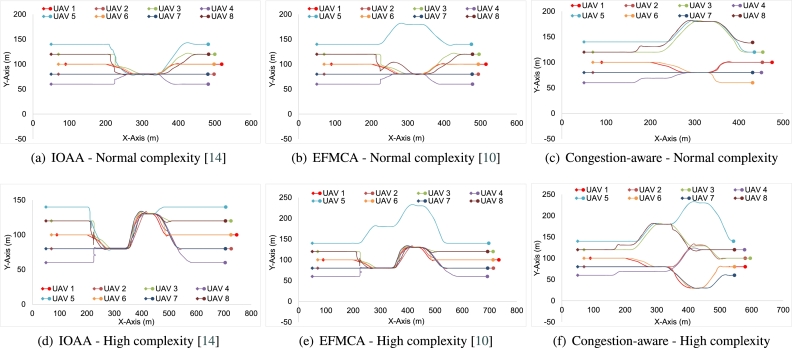
Traces of the overall movement while utilizing three different approaches. Trend of agents' trajectories utilizing (a) leader-follower based approach while navigating through single layer of obstacles, maximum congestion; (b) shortest path approach while navigating through single layer of obstacles, one agent deviates to its respective shortest path, otherwise maximum congestion; (c) the proposed congestion-aware scheme while navigating through single layer of obstacles, minimum congestion; (d) leader-follower based approach while navigating through nested layers of obstacles, maximum congestion; (e) shortest path approach while navigating through nested layers of obstacles, one agent deviates to its respective shortest path, otherwise maximum congestion; (f) the proposed congestion-aware scheme while navigating through nested layers of obstacles, minimum congestion.

**Figure 12:**
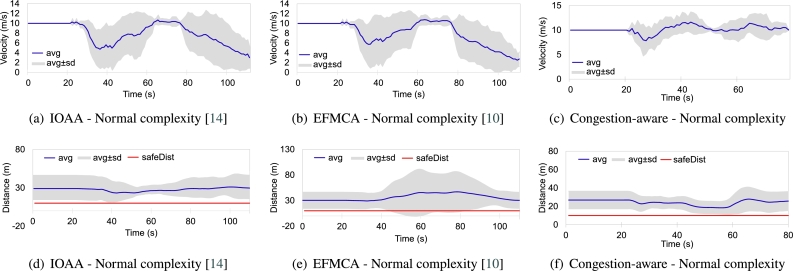
Normal setup scenario comparison of average velocity and distance maintained by the agents, while employing the mentioned three approaches, in the swarm from the start of the mission, while navigating through the obstacles, and towards the end of the mission ± standard deviation.

Firstly, Fig. 11, show the overall trend of the agents while navigating through the set of obstacles while utilizing three different methodologies. Figs. 11(a), 11(b), and 11(c) show the trajectory traces of the agents, in the normal complexity scenario (single layer of obstacles) while utilizing the IOAA, EFMCA, and congestion-aware techniques respectively. As the figures show, maximum delays due to congestion occur in Fig. 11(a) and 11(b), and it took the swarm to get back into formation *t* = 115 and *t* = 110 s respectively. Whereas, in 11(a) congestion is minimized by employing the proposed technique and subsequently, the agents manage to maintain the desired formation shape after avoiding the obstacles at *t* = 80 s. Correspondingly, Fig. 12 shows the average velocity and distance maintained by the swarm as a whole during the mission. The delays due to congestion and as a consequence the variation in the average velocity of the swarm is noticeable in Figs. 12(a) and 12(b), whereas, in comparison it can be seen in Fig. 12(c), average velocity and minimal variance, the proposed congestion-aware approach outperforms the mentioned methodologies. The average distance maintained by the agents with their respective leaders is shown in Figs. 12(d), 12(e), and 12(f). Here, in 12(d) (IOAA) and 12(f) (congestion-aware approach), the distance maintained and the variance is quite linear and closer to the desired defined distances between the agents. However, in 12(e) (EFMCA), due to the fact that agents can break away from formation for their personal benefit, i.e., shortest path avoidance, variance in the distance between the agents is much higher.

Similarly, Figs. 11(d), 11(e), and 11(f) show the overall trajectories of the agents while navigating through the nested layers of obstacles, i.e., high complexity scenario. The first set of obstacles are shifted down relative to the center of the swarm, as illustrated in Fig. 5. As can be seen from Fig. 11(d), that the swarm while utilizing only the leader-follower based approach faces the maximum congestion while navigating through the obstacles. Since the agents just follow their leader(s) and maintain the desired safe distances to avoid colliding with each other, they have to slow down, hover at their respective locations to allow other agents to merge in the queue while morphing the shape for navigating through the obstacles. Fig. 11(e), shows the movement trace of the agents while utilizing the shortest path approach for collision avoidance. As can be seen, besides agent 5, all other agents have their shortest path towards the same gap. This leads to maximum congestion as well. Fig. 11(f) shows the overall behavior of the swarm while utilizing the proposed congestion-aware approach. The setup is the same as the above setups with all agents, except agent 5, having their shortest paths towards the same gap between the obstacles. However, while utilizing the proposed approach, agents 3 and 8, are forced to take the distance penalty in order to get the most optimal solution for the swarm as a whole, i.e., minimum congestion.

**Figure 13:**
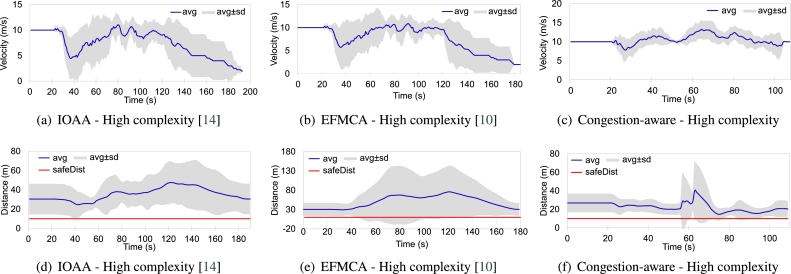
High complexity scenario comparison of average velocity and distance maintained by the agents, while employing the mentioned three approaches, in the swarm from the start of the mission, while navigating through the obstacles, and towards the end of the mission ± standard deviation.

Fig. 13 shows the average velocity of the swarm and the average distances maintained by the agents with their respective leaders, from mission start till the agents come back to the desired formation shape while employing the proposed approach in comparison with the mentioned approaches. As can be seen from Fig. 13(a) the average velocity maintained by the swarm as a whole has major deviations due to the fact that while using only the leader-follower based queuing approach [14], all the agents navigate through the same narrow gap available between the obstacles, resulting in maximum congestion. This forces all the agents to slow down in order to allow other agents to come into the queue while morphing the formation. Moreover, as shown in Fig. 13(b), while utilizing the leader-follower based approach integrated with shortest path avoidance maneuvers [10], the deviation in the average velocity is almost the same as in the previous case with the exception of one agent (UAV 5, shown in Fig. 11(e)). Here the UAV 5 is the only agent that has the shortest path towards a different gap. Whereas, as shown in Fig. 13(c), the swarm's overall velocity remains approximately at the desired/optimal velocity even while navigating through the obstacles. This is due to the fact that, upon utilizing the congestion-aware estimation, the leader of the swarm forces two agents (UAV 3 and 8, shown in Fig. 11(f)) to take distance penalty in order to achieve the optimal solution for the swarm.

**Figure 14:**
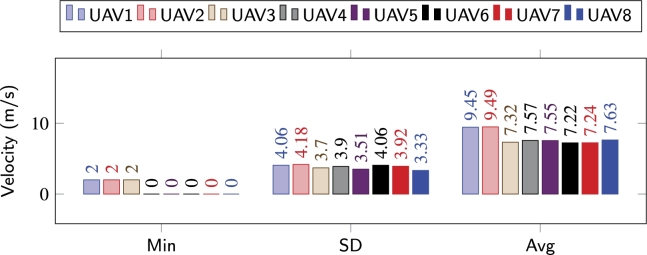
IOAA - High complexity [14].

**Figure 15:**
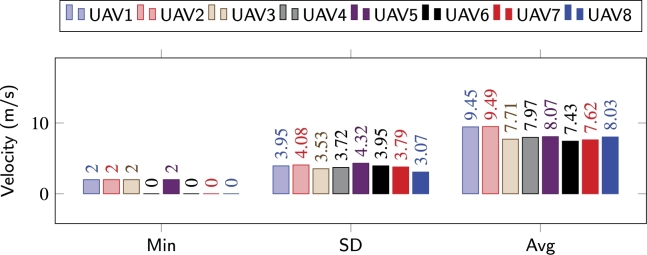
EFMCA - High complexity [10].

**Figure 16:**
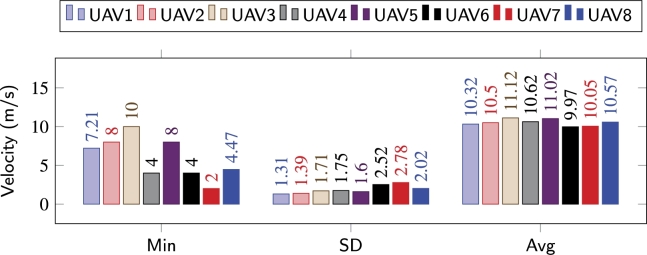
Congestion-aware - High complexity.

Similarly, the average distances maintained by the agents with their respective leaders, is shown in Figs. 13(d), 13(e), and 13(f). Average distances between the agents in [14] and [10] are much higher as compared to the distances maintained while utilizing the congestion-aware approach. This is simply due to the delays that occur due to overpopulation while navigating through the obstacles. Whereas, as shown in Fig. 13(f), the only time the average distance has an exponential peak (at around *t* = 60 s) is during the reformation phase and due to the change of the respective leaders. During all the performed experiments, the average distance never went below, i.e., crossed, the safe distance line, meaning that no collisions occurred in any of the setups. It is important to note here, that with [10] and [14] approaches, due to congestion, the overall time for the swarm to come back into formation increased significantly, and the leaders, upon arriving at their respective coordinates, slowed down to allow the remaining followers to approach them without accelerating aggressively. Velocity change behavior of individual agents/UAVs throughout the mission while employing [10], [14], and the proposed congestion-aware techniques can be analyzed. In Figs. 14 and 15, it can be clearly seen that due to the overpopulation and congestion, several of the agents decelerated to 0 m/s, i.e., came to hovering state, and wait for their turn while creating space for other agents in the swarm to merge in the queue. Whereas it is evident from Fig. 16, since the proposed methodology takes the measures for minimizing congestion, none of the UAV came to hovering state to wait for its turn, only UAV 7 decelerated to 2 m/s, and the overall trend of the UAVs on average is much closer to the optimal navigational velocity. Therefore, utilizing the congestion-aware approach the swarm comes back to the desired formation shape, after evading all the obstacles, in approximately half the time it takes for the other two approaches. Moreover, the deviation in their velocities with [10] and [14] is also higher as compared to deviation while utilizing the proposed technique.

In order to estimate the energy-saving effect of the proposed approach, we first consider the energy consumption of the swarm of eight drones while bypassing a single obstacle, as discussed in section 2 and depicted in Fig. 3. In the following discussion, we use the results of [47], where total power required by a drone weighing 20 Newton, having four blades with a rotor radius of 40 cm is plotted against the drone's flying speed. We have selected the nominal speed as 10 m/s, which is a close approximation of the Maximum Endurance speed calculated by [47]. To further enhance endurance, we perform gradual acceleration and deceleration with maximum speed fixed at 20 m/s, the power consumption is seen to rise drastically at speeds above this value, as indicated in Fig. 2 of [47] and validated within statistical bounds by [48] after extensive experimentation. The total energy consumed to perform a maneuver can be found by integrating the instantaneous powers over the whole flight time, from the start time ($t_{s}$) to the finish time ($t_{f}$). Since our algorithm works in discrete time steps, we calculate each drone's total energy consumption as shown in Eq. (13), and sum up individual results to yield the total energy consumption of the swarm as a whole.

(13) $$E_{total}=\sum_{t=t_{s}}^{t_{f}}P(t)\Delta t$$

**Figure 17:**
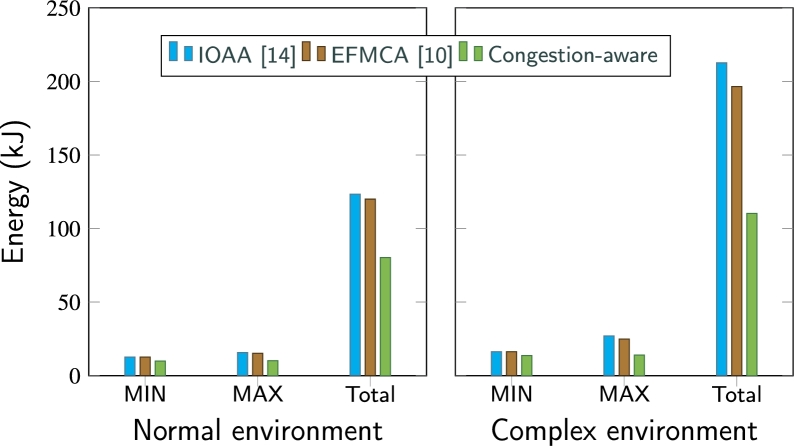
MIN = minimum energy consumed by an individual UAV, MAX = maximum energy consumed by an individual UAV, and Total energy consumption of the swarm for all techniques in normal complexity and high complexity environmental scenarios.

Fig. 17 shows the comparison between the energy consumption of the swarm as a whole for all three experiments in two different complexity levels of the environment. In the normal complexity scenario, with the proposed scheme, the swarm utilized 80.25 kJ from mission start till the agents come back into the intended formation shape after bypassing the obstacles, whereas, employing the traditional approaches IOAA and EFMCA, the swarm consumed 123.34 kJ and 120 kJ respectively. Moreover, employing the proposed approach and bypassing the set of obstacles in high complexity scene, the total energy consumed by the swarm was 110.26 kJ, resulting in 56.11% and 51.8% lower energy consumption as compared to the traditional approaches, i.e., the shortest path method and leader-follower queuing, where the swarm consumed 196.49 kJ and 212.63 kJ respectively.

## 7 Conclusions

In this paper, we present a methodology for finding an optimal solution to (1) avoid the congestion that may happen in a swarm of autonomous agents while avoiding collisions with obstacles and to (2) bring the agents back into the desired formation shape after evading an obstacle. In the proposed method, an agent population control factor is considered in relation to the obstacle(s) in the vicinity, with a time constraint for the disturbance phase. In this approach, the leader of the swarm takes a centralized decision by utilizing the parameters of the agents (coordinates, velocities) and obstacles to find the population factor, and based on that the grouping configuration of the swarm is determined. Then it selects the group setup which provides the shortest overall congestion delay. Afterwards, in the convergence phase of the proposed methodology, thin-plate splines based technique is utilized to optimally bring the agents back into the intended formation by mapping the closest agents to the nearest points of the desired formation. We demonstrated via simulations that by utilizing the congestion control approach we can minimize the delays, save time, and consequently minimize the overall energy consumption of the swarm.

In our future work, we aim to refine the proposed methodology by considering non-negligible computation times and communication delays in calculating realistic reaction distances.

## Declarations

### Author contribution statement

Jawad N. Yasin: Conceived and designed the experiments; Performed the experiments; Analyzed and interpreted the data; Wrote the paper.

Mohammad-Hashem Haghbayan & Muhammad Mehboob Yasin: Conceived and designed the experiments; Analyzed and interpreted the data; Contributed reagents, materials, analysis tools or data; Wrote the paper.

Juha Plosila: Conceived and designed the experiments; Contributed reagents, materials, analysis tools or data; Wrote the paper.

### Declaration of interests statement

The authors declare no conflict of interest.

### Data availability statement

No data was used for the research described in the article

### Funding statement

This work was supported by the 10.13039/501100002341Academy of Finland funded research projects (AURORA: 330493, ADAFI 335512) and the 10.13039/501100004181Nokia Foundation (Award No. 20200147).

### Additional information

No additional information is available for this paper.

## Footnotes

1 Group labeling is done for illustration and explanation purposes. Depending on the scenario, agents can be divided into several groups and subgroup sets {groupSet}.

## References

[1] Hamann H. (2018). Swarm Robotics: a Formal Approach.

[2] Dorigo M., Roosevelt Av F.D. (2004). Special Issue, Autonomous Robots.

[3] McGuire K.N., De Wagter C., Tuyls K., Kappen H.J., de Croon G.C.H.E. (2019). Minimal navigation solution for a swarm of tiny flying robots to explore an unknown environment. Sci. Robot..

[4] Tagliabue A., Kamel M., Verling S., Siegwart R., Nieto J. (2017). Proc. IEEE International Conference on Robotics and Automation.

[5] Shakhatreh H., Sawalmeh A.H., Al-Fuqaha A., Dou Z., Almaita E., Khalil I., Othman N.S., Khreishah A., Guizani M. (2019). Unmanned aerial vehicles (UAVs): a survey on civil applications and key research challenges. IEEE Access.

[6] Grocholsky B., Keller J., Kumar V., Pappas G. (2006). Cooperative air and ground surveillance. IEEE Robot. Autom. Mag..

[7] Alonso-Mora J., Schoch M., Breitenmoser A., Siegwart R., Beardsley P. (2012). Proc. IEEE/RSJ International Conference on Intelligent Robots and Systems.

[8] Mohamed S.A.S., Haghbayan M., Westerlund T., Heikkonen J., Tenhunen H., Plosila J. (2019). A survey on odometry for autonomous navigation systems. IEEE Access.

[9] Nguyen H., Dang T., Alexis K. (2020). Proc. IEEE International Conference on Robotics and Automation.

[10] Yasin J.N., Mohamed S.A.S., Haghbayan M.H., Heikkonen J., Tenhunen H., Yasin M.M., Plosila J. (2020). Energy-efficient formation morphing for collision avoidance in a swarm of drones. IEEE Access.

[11] Yasin J.N., Mohamed S.A.S., Haghbayan M., Heikkonen J., Tenhunen H., Plosila J. (2020). Unmanned aerial vehicles (UAVs): collision avoidance systems and approaches. IEEE Access.

[12] Forootaninia Z., Karamouzas I., Narain R. (2017). Robotics: Science and Systems, vol. 7.

[13] He L., Bai P., Liang X., Zhang J., Wang W. (2018). Feedback formation control of UAV swarm with multiple implicit leaders. Aerosp. Sci. Technol..

[14] Wu X., Wang S., Xing M. (2019). Observer-based leader-following formation control for multi-robot with obstacle avoidance. IEEE Access.

[15] Balch T., Arkin R.C. (Dec 1998). Behavior-based formation control for multirobot teams. IEEE Trans. Robot. Autom..

[16] Lawton J.R.T., Beard R.W., Young B.J. (2003). A decentralized approach to formation maneuvers. IEEE Trans. Robot. Autom..

[17] Dong L., Chen Y., Qu X. (2016). Formation control strategy for nonholonomic intelligent vehicles based on virtual structure and consensus approach. Proc. Eng..

[18] Li N.H.M., Liu H.H.T. (2008). 2008 American Control Conference.

[19] Su H., Wang X., Lin Z. (Feb 2009). Flocking of multi-agents with a virtual leader. IEEE Trans. Autom. Control.

[20] Mostaghim S., Steup C., Witt F. (2016). 2016 IEEE Symposium Series on Computational Intelligence.

[21] Majd A., Loni M., Sahebi G., Daneshtalab M. (Aug 2020). Improving motion safety and efficiency of intelligent autonomous swarm of drones. Drones.

[22] Narayanan K., Honkote V., Ghosh D., Baldev S. (2019). Proc. 32nd International Conference on VLSI Design and 2019 18th International Conference on Embedded Systems.

[23] Yasin J.N., Mohamed S.A.S., Haghbayan M.H., Heikkonen J., Tenhunen H., Plosila J., Demazeau Yves, Holvoet Tom, Corchado Juan M., Costantini Stefania (2020). Advances in Practical Applications of Agents, Multi-Agent Systems, and Trustworthiness. The PAAMS Collection.

[24] Tseng C.M., Chau C.K., Elbassioni K., Khonji M. (2017).

[25] Pereira G.A.S., Choudhury S., Scherer S. (2016). 2016 International Conference on Unmanned Aircraft Systems.

[26] Hsu D., Latombe J.C., Kurniawati H. (2006). On the probabilistic foundations of probabilistic roadmap planning. Int. J. Robot. Res..

[27] Zhang J., Hu C., Chadha R.G., Singh S. (2019). 2019 IEEE/RSJ International Conference on Intelligent Robots and Systems.

[28] Gammell J.D., Srinivasa S.S., Barfoot T.D. (2015). 2015 IEEE International Conference on Robotics and Automation.

[29] LaValle S.M. (2006).

[30] Portugal D., Rocha R. (2010). Proceedings of the 2010 ACM Symposium on Applied Computing.

[31] Iocchi L., Marchetti L., Nardi D. (2011). 2011 IEEE/RSJ International Conference on Intelligent Robots and Systems.

[32] Portugal D., Rocha R.P. (2013). Distributed multi-robot patrol: a scalable and fault-tolerant framework. Robot. Auton. Syst..

[33] Hoshino S., Seki H. (2013). Multi-robot coordination for jams in congested systems. Robot. Auton. Syst..

[34] Sadat S.A., Vaughan R.T. (2010). ALIFE.

[35] Street C., Lacerda B., Mühlig M., Hawes N. (2020). Proceedings of the 19th International Conference on Autonomous Agents and MultiAgent Systems.

[36] Yasin J.N., Mahboob H., Haghbayan M.H., Yasin M.M., Plosila Juha (2021). Energy-efficient navigation of an autonomous swarm with adaptive consciousness. Remote Sens..

[37] Yasin J.N., Mohamed S.A.S., Haghbayan M.H., Heikkonen J., Tenhunen H., Yasin M.M., Plosila J., Arai Kohei (2021). Advances in Information and Communication.

[38] Chand B.N., Mahalakshmi P., Naidu V.P.S. (2017). Proc. International Conference on Electrical, Electronics, Communication, Computer, and Optimization Techniques.

[39] Stolaroff J.K., Samaras C., O'Neill E.R., Lubers A., Mitchell A.S., Ceperley D. (February 2018). Energy use and life cycle greenhouse gas emissions of drones for commercial package delivery. Nat. Commun..

[40] Yasin J.N., Haghbayan M.H., Heikkonen J., Tenhunen H., Plosila J. (2019). Proc. International Symposium on Computer Science and Intelligent Control.

[41] Myronenko A., Song X. (2009). Point-set registration: coherent point drift. arxiv:0905.2635.

[42] Guo P., Hu W., Ren H., Zhang Y. (Oct 2018). Proc. IEEE/RSJ International Conference on Intelligent Robots and Systems.

[43] Chui H., Rangarajan A. (June 2000). Proceedings IEEE Conference on Computer Vision and Pattern Recognition, vol. 2.

[44] Soria E., Schiano F., Floreano D. (2020). Proc. IEEE/RSJ International Conference on Intelligent Robots and Systems.

[45] https://www.digi.com/resources/library/data-sheets/ds_xbeemultipointmodules.

[46] https://pdf.directindustry.com/pdf/velodynelidar/puck-lite-datasheets/182407-676096.html.

[47] Zeng Y., Xu J., Zhang R. (2019). Energy minimization for wireless communication with rotary-wing UAV. IEEE Trans. Wirel. Commun..

[48] Gao N., Zeng Y., Wang J., Wu D., Zhang C., Song Q., Qian J., Jin S. (2020). Energy model for UAV communications: experimental validation and model generalization. arxiv:2005.01305.

